# A symmetric version of the generalized alternating direction method of multipliers for two-block separable convex programming

**DOI:** 10.1186/s13660-017-1405-0

**Published:** 2017-06-05

**Authors:** Jing Liu, Yongrui Duan, Min Sun

**Affiliations:** 1 School of Economics and Management, Tongji University, Shanghai, 200092 P.R. China; 2 School of Data Sciences, Zhejiang University of Finance and Economics, Zhejiang, 310018 P.R. China; 3 School of Mathematics and Statistics, Zaozhuang University, Shandong, 277160 P.R. China; 4 School of Management, Qufu Normal University, Shandong, 276826 P.R. China

**Keywords:** 90C25, 90C30, alternating direction method of multipliers, convex programming, mixed variational inequalities, compressed sensing

## Abstract

This paper introduces a symmetric version of the generalized alternating direction method of multipliers for two-block separable convex programming with linear equality constraints, which inherits the superiorities of the classical alternating direction method of multipliers (ADMM), and which extends the feasible set of the relaxation factor *α* of the generalized ADMM to the infinite interval $[1,+\infty)$. Under the conditions that the objective function is convex and the solution set is nonempty, we establish the convergence results of the proposed method, including the global convergence, the worst-case $\mathcal{O}(1/k)$ convergence rate in both the ergodic and the non-ergodic senses, where *k* denotes the iteration counter. Numerical experiments to decode a sparse signal arising in compressed sensing are included to illustrate the efficiency of the new method.

## Introduction

We consider the two-block separable convex programming with linear equality constraints, where the objective function is the sum of two individual functions with decoupled variables:

1 $$ \min\bigl\{ \theta_{1}(x_{1})+\theta _{2}(x_{2})|A_{1}x_{1}+A_{2}x_{2}=b,x_{1} \in\mathcal{X}_{1},x_{2}\in\mathcal{X}_{2}\bigr\} , $$

where $\theta_{i}:\mathcal{R}^{n_{i}}\rightarrow\mathcal{R}$ ($i=1,2$) are closed proper convex functions; $A_{i}\in\mathcal{R}^{l\times n_{i}}$ ($i=1,2$) and $b\in\mathcal{R}^{l}$, and $\mathcal{X}_{i}\subseteq\mathcal {R}^{n_{i}}$ ($i=1,2$) are given nonempty closed convex sets. The linear constrained convex problem (1) is a unified framework of many problems arising in real world, including compressed sensing, image restoration, and statistical learning, and so forth (see, for example, [1–3]). An important special case of (1) is the following linear inverse problem:

2 $$ \min_{x\in\mathcal{R}^{n}}\mu\|x\|_{1}+ \frac {1}{2}\|Ax-y\|^{2}, $$

where $A\in\mathcal{R}^{m\times n}$ and $y\in\mathcal{R}^{m}$ are given matrix and vector, $\mu>0$ is a regularization parameter and $\|x\| _{1}$ is the $\ell_{1}$-norm of a vector *x* defined as $\|x\|_{1}=\sum_{i=1}^{n}|x_{i}|$. Then setting $x_{1}:=Ax-y$, $x_{2}:=x$, (2) can be converted into the following two-block separable convex programming:

3 $$ \begin{aligned} &\min\frac{1}{2}\|x_{1} \|^{2}+\mu\|x_{2}\|_{1} \\ &\quad \mbox{s.t. } {-}x_{1}+Ax_{2}=y, \\ &\hphantom{\quad \mbox{s.t.} }\ x_{1}\in\mathcal{R}^{m}, x_{2} \in\mathcal{R}^{n}, \end{aligned} $$

which is a special case of problem (1) with the following specifications:

$$\theta_{1}(x_{1}):=\frac{1}{2}\|x_{1} \|^{2}, \qquad \theta_{2}(x_{2}):=\mu \|x_{2}\|_{1},\qquad A_{1}:=-I_{m}, \qquad A_{2}:=A,\qquad b:=y. $$

### Existing algorithms

In their seminal work, Glowinski *et al.* [4] and Gabay *et al.* [5] independently developed the alternating direction method of multipliers (ADMM), which is an influential first-order method for solving problem (1). ADMM can be regarded as an application of the Douglas-Rachford splitting method (DRSM) [6] to the dual of (1), or a special case of the proximal point algorithm (PPA) [7, 8] in the cyclic sense. We refer to [9] for a more detailed relationship. With any initial vectors $x_{2}^{0}\in\mathcal{X}_{2}$, $\lambda ^{0}\in\mathcal{R}^{l}$, the iterative scheme of ADMM reads

4 $$ \textstyle\begin{cases} x_{1}^{k+1}\in\operatorname{argmin}_{x_{1}\in\mathcal{X}_{1}}\{ \theta_{1}(x_{1})-x_{1}^{\top}A_{1}^{\top}\lambda^{k}+\frac{\beta}{2}\| A_{1}x_{1}+A_{2}x_{2}^{k}-b\|^{2}\}, \\ x_{2}^{k+1}\in\operatorname{argmin}_{x_{2}\in\mathcal{X}_{2}}\{\theta _{2}(x_{2})-x_{2}^{\top}A_{2}^{\top}\lambda^{k} +\frac{\beta}{2}\| A_{1}x_{1}^{k+1}+A_{2}x_{2}-b\|^{2}\}, \\ \lambda^{k+1}=\lambda^{k}-\beta (A_{1}x_{1}^{k+1}+A_{2}x_{2}^{k+1}-b), \end{cases} $$

where $\lambda\in\mathcal{R}^{l}$ is the Lagrangian multiplier and $\beta>0$ is a penalty parameter. The main characteristics of ADMM are that it in full exploits the separable structure of problem (1), and that it updates the variables $x_{1}$, $x_{2}$, *λ* in an alternating order by solving a series of low-dimensional sub-problems with only one unknown variable.

In the past few decades, ADMM has received a revived interest, and it has become a research focus in optimization community, especially in the (non)convex optimization. Many efficient ADMM-type methods have been developed, including the proximal ADMM [8, 10], the generalized ADMM [11], the symmetric ADMM [12], the inertial ADMM [13], and some proximal ADMM-type methods [14–18]. Specifically, the proximal ADMM attaches some proximal terms to the sub-problems of ADMM (4). The generalized ADMM updates the variables $x_{2}$ and *λ* by including a relaxation factor $\alpha\in(0,2)$, and $\alpha\in(1,2)$ is often advantageous to speed up its performance. The symmetric ADMM updates the Lagrangian multiplier *λ* twice at each iteration and includes two relaxation factors $\alpha\in(0,1)$, $\beta\in(0,1)$. Recent researches of the symmetric ADMM can be found in [12, 15, 18]. The inertial ADMM unifies the basic ideas of the inertial PPA and ADMM, which utilizes the latest two iterates to generate the new iterate, therefore it can be viewed as a multistep method. For the proximal ADMM, the objective functions of its sub-problems are often strongly convex, which are often easier to be solved than those of (4). However, a new challenge has arisen for the proximal ADMM-type methods. It is how to choose a proper proximal matrix. In fact, most proximal ADMM-type methods need to estimate the matrix norm $\| A_{i}^{\top}A_{i}\|$ ($i=1,2$), which demands lots of calculations, especially for large $n_{i}$ ($i=1,2$). Quite recently, some customized Douglas-Rachford splitting algorithms [19–21], and the proximal ADMM-type methods with indefinite proximal regularization are developed [22, 23], which dissolve the above problem to some extent. All the above mentioned ADMM-type methods are generalizations of the classical ADMM, because they all reduce to the iterative scheme (4) by choosing some special parameters. For more new development of the ADMM-type methods, including the convergence rate, acceleration techniques, its generalization for solving multi-block separable convex programming and nonconvex, nonsmooth programming, we refer to [24–28].

### Contributions and organization

We are going to further study the generalized ADMM. Note that the first sub-problem in the generalized ADMM is irrelevant to the relaxation factor *α*. That is, the updating formula for $x_{1}$ does not incorporate the relaxation factor *α* explicitly. Furthermore, $\alpha\in(1,2)$ is often advantageous for the generalized ADMM [14]. Therefore, in this paper, we are going to propose a new generalized ADMM, whose both sub-problems incorporate the relaxation factor *α* directly. The new method generalizes the method proposed in [29] by relaxing the feasible set of *α* from the interval $[1,2)$ to the infinite interval $[1,+\infty)$, and can be viewed as a symmetric version of the generalized ADMM.

The rest of the paper is organized as follows. In Section 2, we summarize some necessary preliminaries and characterize problem (1) by a mixed variational inequality problem. In Section 3, we describe the new symmetric version of the generalized ADMM and establish its convergence results in detail. In Section 4, some compressed sensing experiments are given to illustrate the efficiency of the proposed method. Some conclusions are drawn in Section 5.

## Preliminaries

In this section, some necessary preliminaries which are useful for further discussions are presented, and to make our analysis more succinct, some positive definite or positive semi-definite block matrices are defined and their properties are investigated.

For two real matrices $A\in\mathcal{R}^{s\times m}$, $B\in\mathcal {R}^{n\times s}$, the Kronecker product of *A* and *B* is defined as $A\otimes B=(a_{ij}B)$. Let $\|\cdot\|_{p}$ ($p = 1, 2$) denote the standard definition of $\ell_{p}$-norm; in particular, $\|\cdot\|=\|\cdot\|_{2}$. For any two vectors $x,y\in\mathcal {R}^{n}$, $\langle x,y\rangle$ or $x^{\top}y$ denote their inner product, and for any symmetric matrix $G\in\mathcal{R}^{n\times n}$, the symbol $G\succ0$ (resp., $G\succeq0$) denotes that *G* is positive definite (resp., semi-definite). For any $x\in\mathcal{R}^{n}$ and $G\succeq0$, the *G*-norm $\|x\|_{G}$ of the vector *x* is defined as $\sqrt{x^{\top}Gx}$. The effective domain of a closed proper function $f: \mathcal{X} \rightarrow(-\infty,+\infty]$ is defined as $\operatorname{dom}(f):= \{x\in\mathcal{X}|f(x) < +\infty\}$, and the symbol $\operatorname{ri}(\mathcal{C})$ denotes the set of all relative interior points of a given nonempty convex set $\mathcal{C}$. Furthermore, we use the following notations:

$$x=(x_{1},x_{2}),\qquad w=(x,\lambda). $$

### Definition 2.1

[30]

A function $f:\mathcal {R}^{n}\rightarrow\mathcal{R}$ is convex if and only if

$$f\bigl(\alpha x+(1-\alpha)y\bigr)\leq\alpha f(x)+(1-\alpha)f(y),\quad \forall x,y\in \mathcal{R}^{n}, \alpha\in[0,1]. $$

Then, for a convex function $f:\mathcal{R}^{n}\rightarrow\mathcal{R}$, we have the following basic inequality:

5 $$ f(x)\geq f(y)+\langle\xi, x-y\rangle,\quad \forall x,y\in \mathcal{R}^{n}, \xi\in\partial f(y), $$

where $\partial f(y)=\{\xi\in\mathcal{R}^{n}:f(\bar{y})\geq f(y)+\langle \xi,\bar{y}-y\rangle, \mbox{for all } \bar{y}\in\mathcal{R}^{n}\}$ denotes the subdifferential of $f(\cdot)$ at the point *y*.

Throughout the paper, we make the following standard assumptions for problem (1).

### Assumption 2.1

The functions $\theta_{i}(\cdot)$ ($i=1,2$) are convex.

### Assumption 2.2

The matrices $A_{i}$ ($i=1,2$) are full-column rank.

### Assumption 2.3

The generalized Slater condition holds, *i.e.*, there is a point $(\hat{x}_{1},\hat{x}_{2})\in\operatorname{ri}(\operatorname{dom}\theta_{1}\times\operatorname{dom}\theta_{2})\cap\{x=(x_{1},x_{2})\in\mathcal {X}_{1}\times\mathcal{X}_{2}| A_{1}{x}_{1}+A_{2}{x}_{2}=b\}$.

### The mixed variational inequality problem

Under Assumption 2.3, it follows from Theorem 3.22 and Theorem 3.23 of [31] that $x^{*}=({x}_{1}^{*},{x}_{2}^{*})\in\mathcal{R}^{n_{1}+n_{2}}$ is an optimal solution to problem (1) if and only if there exists a vector $\lambda^{*}\in\mathcal{R}^{l}$ such that $({x}_{1}^{*},{x}_{2}^{*},\lambda^{*})$ is a solution of the following KKT system:

6 $$ \textstyle\begin{cases} 0\in\partial\theta_{i}(x_{i}^{*})-A_{i}^{\top}\lambda^{*}+N_{\mathcal {X}_{i}}(x_{i}^{*}),\quad i=1,2, \\ A_{1}x_{1}^{*}+A_{2}x_{2}^{*}=b, \end{cases} $$

where $N_{\mathcal{X}_{i}}(x_{i}^{*})$ is the normal cone of the convex set $\mathcal{X}_{i}$ at the point $x_{i}^{*}$, which is defined as $N_{\mathcal{X}_{i}}(x_{i}^{*})=\{z\in\mathcal{R}^{n_{i}}|\langle z,x_{i}-x_{i}^{*}\rangle\leq0, \forall x_{i}\in\mathcal{X}_{i}\}$. Then, for the nonempty convex set $\mathcal{X}_{i}$ and $\forall x_{i}\in \mathcal{X}_{i}$, it follows from [32] (Example 2.123) that $N_{\mathcal{X}_{i}}(x_{i})=\partial\delta(\cdot|\mathcal{X}_{i})(x_{i})$, where $\delta(\cdot|\mathcal{X}_{i})$ is the indicator function of the set $\mathcal{X}_{i}$, and $\partial\delta(\cdot|\mathcal{X}_{i})(x_{i})$ is the subdifferential mappings of $\delta(\cdot|\mathcal{X}_{i})$ at the point $x_{i}\in\mathcal{X}_{i}$.

#### Lemma 2.1

*For any vector* $x_{i}^{*}\in\mathcal{R}^{n_{i}}$, $\lambda ^{*}\in\mathcal{R}^{l}$, *the relationship* $0\in\partial\theta_{i}(x_{i}^{*})-A_{i}^{\top}\lambda^{*}+\partial\delta(\cdot |\mathcal{X}_{i})(x_{i}^{*})$ *is equivalent to* $x_{i}^{*}\in\mathcal{X}_{i}$ *and the inequality*

$$\theta_{i}(x_{i})-\theta_{i} \bigl(x_{i}^{*}\bigr)+\bigl(x_{i}-x_{i}^{*} \bigr)^{\top}\bigl(-A_{i}^{\top}\lambda^{*}\bigr)\geq 0, \quad \forall x_{i}\in\mathcal{X}_{i}. $$

#### Proof

From $0\in\partial\theta_{i}(x_{i}^{*})-A_{i}^{\top}\lambda ^{*}+\partial\delta(\cdot|\mathcal{X}_{i})(x_{i}^{*})$, we have $x_{i}^{*}\in \mathcal{X}_{i}$ and there exists $\eta_{i}\in\partial\delta(\cdot|\mathcal {X}_{i})(x_{i}^{*})$ such that

$$A_{i}^{\top}\lambda^{*}-\eta_{i}\in\partial \theta_{i}\bigl(x_{i}^{*}\bigr). $$

From the subgradient inequality (5), one has

$$\theta_{i}(x_{i})-\theta_{i} \bigl(x_{i}^{*}\bigr)\geq\bigl(x_{i}-x_{i}^{*} \bigr)^{\top}\bigl(A_{i}^{\top}\lambda^{*}-\eta _{i}\bigr),\quad \forall x_{i}\in\mathcal{R}^{n_{i}}. $$

Thus,

$$\theta_{i}(x_{i})-\theta_{i} \bigl(x_{i}^{*}\bigr)-\bigl(x_{i}-x_{i}^{*} \bigr)^{\top}\bigl(-A_{i}^{\top}\lambda^{*}\bigr)\geq \bigl(x_{i}-x_{i}^{*}\bigr)^{\top}(- \eta_{i})\geq0,\quad \forall x_{i}\in\mathcal{X}_{i}, $$

where the second inequality comes from $x_{i}^{*}\in\mathcal{X}_{i}$ and $\eta_{i}\in\partial\delta(\cdot|\mathcal{X}_{i})(x_{i}^{*})$.

Conversely, from $\theta_{i}(x_{i})-\theta_{i}(x_{i}^{*})+(x_{i}-x_{i}^{*})^{\top}(-A_{i}^{\top}\lambda^{*})\geq0$, $\forall x_{i}\in\mathcal{X}_{i}$, we have

$$\theta_{i}(x_{i})+x_{i}^{\top}\bigl(-A_{i}^{\top}\lambda^{*}\bigr)\geq\theta _{i} \bigl(x_{i}^{*}\bigr)+\bigl(x_{i}^{*}\bigr)^{\top}\bigl(-A_{i}^{\top}\lambda^{*}\bigr),\quad \forall x_{i}\in\mathcal{X}_{i}, $$

which together with $x_{i}^{*}\in\mathcal{X}_{i}$ implies that

$$x_{i}^{*}=\mathop{\operatorname{argmin}}_{x_{i}\in\mathcal{X}_{i}}\bigl\{ \theta_{i}(x_{i})+x_{i}^{\top}\bigl(-A_{i}^{\top}\lambda^{*}\bigr)\bigr\} . $$

From this and Theorem 3.22 of [31], we have $0\in\partial \theta_{i}(x_{i}^{*})-A_{i}^{\top}\lambda^{*}+\partial\delta(\cdot|\mathcal{X}_{i})(x_{i}^{*})$. This completes the proof. □

#### Remark 2.1

Based on (6) and Lemma 2.1, the vector $x^{*}=({x}_{1}^{*},{x}_{2}^{*})\in\mathcal{R}^{n_{1}+n_{2}}$ is an optimal solution to problem (1) if and only if there exists a vector $\lambda ^{*}\in\mathcal{R}^{l}$ such that

7 $$ \textstyle\begin{cases} ({x}_{1}^{*},{x}_{2}^{*})\in\mathcal{X}_{1}\times\mathcal{X}_{2}; \\ \theta_{i}(x_{i})-\theta_{i}(x_{i}^{*})+(x_{i}-x_{i}^{*})^{\top}(-A_{i}^{\top}\lambda^{*})\geq 0,\quad \forall x_{i}\in\mathcal{X}_{i}, i=1,2; \\ A_{1}x_{1}^{*}+A_{2}x_{2}^{*}=b. \end{cases} $$

Moreover, any $\lambda^{*}\in\mathcal{R}^{l}$ satisfying (7) is an optimal solution to the dual of problem (1). Obviously, (7) can be written as the following mixed variational inequality problem, denoted by $\operatorname{VI}(\mathcal{W},F,\theta)$: Find a vector $w^{*}\in\mathcal{W}$ such that

8 $$ \theta(x)-\theta\bigl(x^{*}\bigr)+\bigl(w-w^{*}\bigr)^{\top}F \bigl(w^{*}\bigr)\geq0,\quad \forall w\in\mathcal{W}, $$

where $\theta(x)=\theta_{1}(x_{1})+\theta_{2}(x_{2})$, $\mathcal{W}=\mathcal {X}_{1}\times\mathcal{X}_{2}\times\mathcal{R}^{l}$, and

9 $$ F(w):=\left ( \textstyle\begin{array}{@{}c@{}} -A_{1}^{\top}\lambda\\ -A_{2}^{\top}\lambda\\ A_{1}x_{1}+A_{2}x_{2}-b \end{array}\displaystyle \right )=\left ( \textstyle\begin{array}{@{}c@{\quad}c@{\quad}c@{}}0&0&-A_{1}^{\top}\\ 0&0&-A_{2}^{\top}\\ A_{1}&A_{2}&0 \end{array}\displaystyle \right )\left ( \textstyle\begin{array}{@{}c@{}}x_{1}\\ x_{2}\\ \lambda \end{array}\displaystyle \right )-\left ( \textstyle\begin{array}{@{}c@{}}0\\ 0\\ b \end{array}\displaystyle \right ). $$

The solution set of $\operatorname{VI}(\mathcal{W},F,\theta)$, denoted by $\mathcal {W}^{*}$, is nonempty by Assumption 2.3 and Remark 2.1. It is easy to verify that the linear function $F(\cdot)$ is not only monotone but also satisfies the following desired property:

$$\bigl(w'-w\bigr)^{\top}\bigl(F\bigl(w' \bigr)-F(w)\bigr)=0,\quad \forall w',w\in\mathcal{W}. $$

### Three matrices and their properties

To present our analysis in a compact way, now let us define some matrices. For any $R_{i}\in\mathcal{R}^{n_{i}\times n_{i}}\ (i=1,2)\succeq0$, set

10 $$ M=\left ( \textstyle\begin{array}{@{}c@{\quad}c@{\quad}c@{}} I_{n_{1}}&0&0\\ 0&I_{n_{2}}&0\\ 0&-\beta A_{2}&I_{l} \end{array}\displaystyle \right ) $$

and for $\alpha\in[1,{+\infty})$, set

11 $$ \begin{aligned} &Q=\left ( \textstyle\begin{array}{@{}c@{\quad}c@{\quad}c@{}} R_{1}&0&0\\ 0&R_{2}+(2\alpha-1)\beta A_{2}^{\top}A_{2}&\frac{1-\alpha}{\alpha }A_{2}^{\top}\\ 0&-A_{2}&\frac{1}{\alpha\beta}I_{l} \end{array}\displaystyle \right ), \\ &H= \left ( \textstyle\begin{array}{@{}c@{\quad}c@{\quad}c@{}} R_{1}&0&0\\ 0&R_{2}+\frac{2\alpha^{2}-2\alpha+1}{\alpha}\beta A_{2}^{\top}A_{2}&\frac{1-\alpha}{\alpha}A_{2}^{\top}\\ 0&\frac{1-\alpha}{\alpha}A_{2}&\frac{1}{\alpha\beta}I_{l} \end{array}\displaystyle \right ). \end{aligned} $$

The above defined three matrices *M*, *Q*, *H* satisfy the following properties.

#### Lemma 2.2

*If* $\alpha\in\mathcal{R}$ *and* $R_{i}\succeq0$ ($i=1,2$), *then the matrix* *H* *defined in* (11) *is positive semi*-*definite*.

#### Proof

Set $t=2\alpha^{2}-2\alpha+1$, which is positive for any $\alpha\in\mathcal{R}$. By (11), we have

$$H=\left ( \textstyle\begin{array}{@{}c@{\quad}c@{\quad}c@{}} R_{1}&0&0\\ 0&R_{2}&0\\ 0&0&0 \end{array}\displaystyle \right )+\left ( \textstyle\begin{array}{@{}c@{\quad}c@{\quad}c@{}} 0&0&0\\ 0&\frac{{t}\beta}{\alpha} A_{2}^{\top}A_{2}&\frac{1-\alpha}{\alpha }A_{2}^{\top}\\ 0&\frac{1-\alpha}{\alpha}A_{2}&\frac{1}{\alpha\beta}I_{l} \end{array}\displaystyle \right ). $$

Obviously, the first part is positive semi-definite, and we only need to prove the second part is also positive semi-definite. In fact, it can written as

$$\frac{1}{\alpha} \left ( \textstyle\begin{array}{@{}c@{\quad}c@{\quad}c@{}} 0&0&0\\ 0&\sqrt{\beta}A_{2}^{\top}&0\\ 0&0&\frac{1}{\sqrt{\beta}}I_{l} \end{array}\displaystyle \right )\left ( \textstyle\begin{array}{@{}c@{\quad}c@{\quad}c@{}} 0&0&0\\ 0&tI_{l}&(1-\alpha)I_{l}\\ 0&(1-\alpha)I_{l}&I_{l} \end{array}\displaystyle \right )\left ( \textstyle\begin{array}{@{}c@{\quad}c@{\quad}c@{}} 0&0&0\\ 0&\sqrt{\beta}A_{2}&0\\ 0&0&\frac{1}{\sqrt{\beta}}I_{l} \end{array}\displaystyle \right ). $$

The middle matrix in the above expression can be further written as

$$\left ( \textstyle\begin{array}{@{}c@{\quad}c@{\quad}c@{}} 0&0&0\\ 0&t&1-\alpha\\ 0&1-\alpha&1 \end{array}\displaystyle \right )\otimes I_{l}, $$

where ⊗ denotes the matrix Kronecker product. The matrix Kronecker product has a nice property: for any two matrices *X* and *Y*, the eigenvalue of $X\otimes Y$ equals the product of $\lambda (X)\lambda(Y)$, where $\lambda(X)$ and $\lambda(Y)$ are the eigenvalues of *X* and *Y*, respectively. Therefore, we only need to show the 2-by-2 matrix

$$\left ( \textstyle\begin{array}{@{}c@{\quad}c@{}} t&1-\alpha\\ 1-\alpha&1 \end{array}\displaystyle \right ) $$

is positive semi-definite. In fact,

$$t-(1-\alpha)^{2}=\alpha^{2}\geq0. $$

Therefore, the matrix *H* is positive semi-definite. The proof is then complete. □

#### Lemma 2.3

*If* $\alpha\in[1,+\infty)$ *and* $R_{i}\succeq0$ ($i=1,2$), *then the matrices* *M*, *Q*, *H* *defined*, *respectively*, *in* (10), (11) *satisfy the following relationships*:

12 $$ HM=Q $$

*and*

13 $$ Q^{\top}+Q-M^{\top}HM\succeq\frac{\alpha -1}{2\alpha}M^{\top}HM. $$

#### Proof

From (10) and (11), we have

$$\begin{aligned} HM =&\left ( \textstyle\begin{array}{@{}c@{\quad}c@{\quad}c@{}} R_{1}&0&0\\ 0&R_{2}+\frac{2\alpha^{2}-2\alpha+1}{\alpha}\beta A_{2}^{\top}A_{2}&\frac{1-\alpha}{\alpha}A_{2}^{\top}\\ 0&\frac{1-\alpha}{\alpha}A_{2}&\frac{1}{\alpha\beta}I_{l} \end{array}\displaystyle \right )\left ( \textstyle\begin{array}{@{}c@{\quad}c@{\quad}c@{}}I_{n_{1}}&0&0\\ 0&I_{n_{2}}&0\\ 0&-\beta A_{2}&I_{l} \end{array}\displaystyle \right ) \\ =&\left ( \textstyle\begin{array}{@{}c@{\quad}c@{\quad}c@{}} R_{1}&0&0\\ 0&R_{2}+(2\alpha-1)\beta A_{2}^{\top}A_{2}&\frac{1-\alpha}{\alpha }A_{2}^{\top}\\ 0&-A_{2}&\frac{1}{\alpha\beta}I_{l} \end{array}\displaystyle \right )=Q. \end{aligned}$$

Then the first assertion is proved. For (13), by some simple manipulations, we obtain

$$\begin{aligned} M^{\top}HM =&M^{\top}Q \\ =&\left ( \textstyle\begin{array}{@{}c@{\quad}c@{\quad}c@{}}I_{n_{1}}&0&0\\ 0&I_{n_{2}}&-\beta A_{2}^{\top}\\ 0&0&I_{l} \end{array}\displaystyle \right )\left ( \textstyle\begin{array}{@{}c@{\quad}c@{\quad}c@{}} R_{1}&0&0\\ 0&R_{2}+(2\alpha-1)\beta A_{2}^{\top}A_{2}&\frac{1-\alpha}{\alpha }A_{2}^{\top}\\ 0&-A_{2}&\frac{1}{\alpha\beta}I_{l} \end{array}\displaystyle \right ) \\ =&\left ( \textstyle\begin{array}{@{}c@{\quad}c@{\quad}c@{}} R_{1}&0&0\\ 0&R_{2}+2\alpha\beta A_{2}^{\top}A_{2}&-A_{2}^{\top}\\ {0}&{-}A_{2}&\frac{1}{\alpha\beta}I_{l} \end{array}\displaystyle \right ). \end{aligned}$$

We now break up the proof into two cases. First, if $\alpha=1$, then

$$\bigl(Q^{\top}+Q\bigr)-M^{\top}HM =\left ( \textstyle\begin{array}{@{}c@{\quad}c@{\quad}c@{}} R_{1}&0&0\\ 0&R_{2}&0\\ 0&0&\frac{1}{\beta}I_{l} \end{array}\displaystyle \right )\succeq0. $$

Therefore, (13) holds. Second, if $\alpha\in(1,+\infty)$, then

14 $$\begin{aligned}& \bigl(Q^{\top}+Q\bigr)-M^{\top}HM \\& \quad =\left ( \textstyle\begin{array}{@{}c@{\quad}c@{\quad}c@{}} R_{1}&0&0\\ 0&R_{2}+(2\alpha-2)\beta A_{2}^{\top}A_{2}&\frac{1-\alpha}{\alpha }A_{2}^{\top}\\ 0&\frac{1-\alpha}{\alpha}A_{2}&\frac{1}{\alpha\beta}I_{l} \end{array}\displaystyle \right ) \\& \quad =\left ( \textstyle\begin{array}{@{}c@{\quad}c@{\quad}c@{}} R_{1}&0&0\\ 0&R_{2}&0\\ 0&0&0 \end{array}\displaystyle \right )+(2\alpha-2)\left ( \textstyle\begin{array}{@{}c@{\quad}c@{\quad}c@{}} 0&0&0\\ 0&\beta A_{2}^{\top}A_{2}&-\frac{1}{2\alpha}A_{2}^{\top}\\ 0&-\frac{1}{2\alpha}A_{2}&\frac{1}{\alpha\beta(2\alpha-2)}I_{l} \end{array}\displaystyle \right ). \end{aligned}$$

Note that

15 $$\begin{aligned}& 4\alpha \left ( \textstyle\begin{array}{@{}c@{\quad}c@{}} \beta A_{2}^{\top}A_{2}&-\frac{1}{2\alpha}A_{2}^{\top}\\ -\frac{1}{2\alpha}A_{2}&\frac{1}{\alpha\beta(2\alpha-2)}I_{l} \end{array}\displaystyle \right )-\left ( \textstyle\begin{array}{@{}c@{\quad}c@{}} 2\alpha\beta A_{2}^{\top}A_{2}&-A_{2}^{\top}\\ -A_{2}&\frac{1}{\alpha\beta}I_{l} \end{array}\displaystyle \right ) \\& \quad =\left ( \textstyle\begin{array}{@{}c@{\quad}c@{}} 2\alpha\beta A_{2}^{\top}A_{2}&-A_{2}^{\top}\\ -A_{2}&\frac{\alpha+1}{\alpha\beta(\alpha-1)}I_{l} \end{array}\displaystyle \right ) \\& \quad =\left ( \textstyle\begin{array}{@{}c@{\quad}c@{}} \sqrt{\beta}A_{2}^{\top}&0\\ 0&\frac{1}{\sqrt{\beta}}I_{l} \end{array}\displaystyle \right )\left ( \textstyle\begin{array}{@{}c@{\quad}c@{}} 2\alpha I_{l}&-I_{l}\\ -I_{l}&\frac{\alpha+1}{\alpha(\alpha-1)}I_{l} \end{array}\displaystyle \right )\left ( \textstyle\begin{array}{@{}c@{\quad}c@{}} \sqrt{\beta}A_{2}&0\\ 0&\frac{1}{\sqrt{\beta}}I_{l} \end{array}\displaystyle \right ). \end{aligned}$$

The middle matrix in the above expression can be further written as

$$\left ( \textstyle\begin{array}{@{}c@{\quad}c@{}} 2\alpha&-1\\ -1&\frac{\alpha+1}{\alpha(\alpha-1)} \end{array}\displaystyle \right )\otimes I_{l}. $$

Since

$$\left ( \textstyle\begin{array}{@{}c@{\quad}c@{}} 2\alpha&-1\\ -1&\frac{\alpha+1}{\alpha(\alpha-1)} \end{array}\displaystyle \right )\succeq0,\quad \forall \alpha>1, $$

the right-hand side of (15) is also positive semi-definite. Thus, we have

16 $$ \left ( \textstyle\begin{array}{@{}c@{\quad}c@{}} \beta A_{2}^{\top}A_{2}&-\frac{1}{2\alpha}A_{2}^{\top}\\ -\frac{1}{2\alpha}A_{2}&\frac{1}{\alpha\beta(2\alpha-2)}I_{l} \end{array}\displaystyle \right )\succeq \frac{1}{4\alpha} \left ( \textstyle\begin{array}{@{}c@{\quad}c@{}} 2\alpha\beta A_{2}^{\top}A_{2}&-A_{2}^{\top}\\ -A_{2}&\frac{1}{\alpha\beta}I_{l} \end{array}\displaystyle \right ). $$

Substituting (16) into (14) and by the expression of $M^{\top}HM$, we obtain (13). The lemma is proved. □

## Algorithm and convergence results

In this section, we first describe the symmetric version of the generalized alternating direction method of multipliers (SGADMM) for $\operatorname{VI}(\mathcal{W},F,\theta)$ formally, and then we prove its global convergence in a contraction perspective and establish its worst-case $\mathcal{O}(1/k)$ convergence rate in both the ergodic and the non-ergodic senses step by step, where *k* denotes the iteration counter.

### Algorithm

#### Algorithm 3.1

SGADMM

Step 0.: Choose the parameters $\alpha\in[1,+\infty)$, $\beta >0$, $R_{i}\in\mathcal{R}^{n_{i}\times n_{i}}\succeq0$ ($i=1,2$), the tolerance $\varepsilon>0$ and the initial iterate $(x_{1}^{0},x_{2}^{0},\lambda^{0})\in\mathcal{X}_{1}\times \mathcal{X}_{2}\times\mathcal{R}^{l}$. Set $k:=0$.
Step 1.: Generate the new iterate ${w}^{k+1}=({x}_{1}^{k+1},{x}_{2}^{k+1},{\lambda}^{k+1})$ by
  17 $$ \textstyle\begin{cases} x_{1}^{k+1}\in\operatorname{argmin}_{x_{1}\in\mathcal{X}_{1}}\{ \theta_{1}(x_{1})-x_{1}^{\top}A_{1}^{\top}\lambda^{k}+\frac{\alpha\beta}{2}\| A_{1}x_{1}+A_{2}x_{2}^{k}-b\|^{2} \\ \hphantom{x_{1}^{k+1}\in{}}{}+\frac{1}{2}\|x_{1}-x_{1}^{k}\|_{R_{1}}^{2}\}, \\ x_{2}^{k+1}\in\operatorname{argmin}_{x_{2}\in\mathcal{X}_{2}}\{\theta _{2}(x_{2})-x_{2}^{\top}A_{2}^{\top}\lambda^{k}+\frac{(2\alpha-1)\beta}{2}\| A_{1}x_{1}^{k+1}+A_{2}x_{2}-b\|^{2} \\ \hphantom{x_{2}^{k+1}\in{}}{}+\frac{1}{2}\|x_{2}-x_{2}^{k}\|_{R_{2}}^{2}\}, \\ \lambda^{k+1}=\lambda^{k}-\beta[\alpha A_{1}x_{1}^{k+1}-(1-\alpha )(A_{2}x_{2}^{k}-b)+A_{2}x_{2}^{k+1}-b]. \end{cases} $$
Step 2.: If
  18 $$ \max\bigl\{ \bigl\Vert R_{1}x_{1}^{k}-R_{1}{x}_{1}^{k+1} \bigr\Vert , \bigl\Vert R_{2}x_{2}^{k}-R_{2}{x}_{2}^{k+1} \bigr\Vert , \bigl\Vert A_{2}x_{2}^{k}-A_{2}{x}_{2}^{k+1} \bigr\Vert , \bigl\Vert \lambda ^{k}-{\lambda}^{k+1} \bigr\Vert \bigr\} < \varepsilon, $$
  then stop and return an approximate solution $({x}_{1}^{k+1},{x}_{2}^{k+1},{\lambda}^{k+1})$ of $\operatorname{VI}(\mathcal{W},F,\theta )$; else set $k:=k+1$, and goto Step 1.

#### Remark 3.1

Obviously, the iterative scheme (17) reduces to the generalized ADMM when $\alpha=1$, and further reduces to (4) when $R_{i}=0$ ($i=1,2$). That is to say, if the parameters $\alpha=1$ and $R_{i}=0$ ($i=1,2$), then the classical ADMM is recovered. Since the convergence results of the (proximal) ADMM have been established in the literature [23, 33, 34], in the following, we only consider $\alpha\in (1,+\infty)$.

### Global convergence

For further analysis, we need to define an auxiliary sequence $\{\hat {w}^{k}\}$ as follows:

19 $$ \hat{w}^{k}=\left ( \textstyle\begin{array}{@{}c@{}}\hat{x}_{1}^{k}\\ \hat{x}_{2}^{k}\\ \hat{\lambda}^{k} \end{array}\displaystyle \right )=\left ( \textstyle\begin{array}{@{}c@{}}{x}_{1}^{k+1}\\ {x}_{2}^{k+1}\\ \lambda^{k}-\alpha\beta (A_{1}{x}_{1}^{k+1}+A_{2}x_{2}^{k}-b) \end{array}\displaystyle \right ). $$

#### Lemma 3.1

*Let* $\{{\lambda}^{k+1}\}$ *and* $\{\hat{\lambda}^{k}\}$ *be the two sequences generated by SGADMM*. *Then*

20 $$ {\lambda}^{k+1}=\hat{\lambda}^{k}-\beta \bigl(A_{2}\hat{x}_{2}^{k}-A_{2}x_{2}^{k} \bigr) $$

*and*

21 $$ \hat{\lambda}^{k}- \biggl( \frac{1}{\alpha}-1 \biggr) \bigl(\hat{\lambda}^{k}- \lambda^{k}\bigr)=\lambda^{k}-(2\alpha-1)\beta\bigl( A_{1}\hat {x}_{1}^{k}+A_{2}{x}_{2}^{k}-b \bigr). $$

#### Proof

From the definition of ${\lambda}^{k+1}$, we get

$$\begin{aligned} {\lambda}^{k+1} =&\lambda^{k}-\beta\bigl[\alpha A_{1}\hat{x}_{1}^{k}-(1-\alpha ) \bigl(A_{2}x_{2}^{k}-b\bigr)+A_{2} \hat{x}_{2}^{k}-b\bigr] \\ =&\lambda^{k}-\beta\bigl[\alpha\bigl(A_{1} \hat{x}_{1}^{k}+A_{2}x_{2}^{k}-b \bigr)+\bigl(A_{2}\hat {x}_{2}^{k}-A_{2}x_{2}^{k} \bigr)\bigr] \\ =&\hat{\lambda}^{k}-\beta\bigl(A_{2}\hat{x}_{2}^{k}-A_{2}x_{2}^{k} \bigr). \end{aligned}$$

Then (20) is proved. For (21), we have

$$\begin{aligned}& \hat{\lambda}^{k}- \biggl( \frac{1}{\alpha}-1 \biggr) \bigl(\hat{ \lambda}^{k}-\lambda^{k}\bigr) \\& \quad = \lambda^{k}-\alpha\beta\bigl(A_{1} \hat{x}_{1}^{k}+A_{2}x_{2}^{k}-b \bigr)+ \biggl( \frac{1}{\alpha}-1 \biggr)\alpha\beta\bigl(A_{1} \hat{x}_{1}^{k}+A_{2}x_{2}^{k}-b \bigr) \\& \quad = \lambda^{k}-(2\alpha-1)\beta\bigl(A_{1}{ \hat{x}}_{1}^{k}+A_{2}x_{2}^{k}-b \bigr). \end{aligned}$$

Therefore (21) is also right. This completes the proof. □

Thus, based on (19) and (20), the two sequences $\{ w^{k}\}$ and $\{\hat{w}^{k}\}$ satisfies the following relationship:

22 $$ {w}^{k+1}=w^{k}-M\bigl(w^{k}- \hat{w}^{k}\bigr), $$

where *M* is defined in (10).

The following lemma shows that the stopping criterion (18) of SGADMM is reasonable.

#### Lemma 3.2

*If* $R_{i}x_{i}^{k}=R_{i}{x}_{i}^{k+1}$ ($i=1,2$), $A_{2}x_{2}^{k}=A_{2}{x}_{2}^{k+1}$ *and* $\lambda^{k}={\lambda}^{k+1}$, *then the iterate* $\hat{w}^{k}=(\hat{x}_{1}^{k},\hat{x}_{2}^{k},\hat{\lambda}^{k})$ *produced by SGADMM is a solution of* $\operatorname{VI}(\mathcal{W},F,\theta)$.

#### Proof

By invoking the optimality condition of the three sub-problems in (4), we have the following mixed variational inequality problems: for any $w=(x_{1},x_{2},\lambda)\in\mathcal{W}$,

$$\textstyle\begin{cases} \theta_{1}(x_{1})-\theta_{1}(\hat{x}_{1}^{k})+(x_{1}-\hat{x}_{1}^{k})^{\top}\{ -A_{1}^{\top}[\lambda^{k}-\alpha\beta(A_{1}\hat {x}_{1}^{k}+A_{2}x_{2}^{k}-b)]+R_{1}{(\hat{x}_{1}^{k} -x_{1}^{k})} \}\geq0, \\ \theta_{2}(x_{2})-\theta_{2}(\hat{x}_{2}^{k})+(x_{2}-\hat{x}_{2}^{k})^{\top}\{ -A_{2}^{\top}[\lambda^{k}-(2\alpha-1)\beta( A_{1}\hat {x}_{1}^{k}+A_{2}\hat{x}_{2}^{k}-b)] \} \\ \quad {}+R_{2}{(\hat{x}_{2}^{k} -x_{2}^{k})}\geq0, \\ (\lambda-\hat{\lambda}^{k})^{\top}[\alpha A_{1}\hat{x}_{1}^{k}-(1-\alpha )(A_{2}x_{2}^{k}-b)+A_{2}\hat{x}_{2}^{k}-b-(\lambda^{k}-{\lambda}^{k+1})/\beta]\geq0. \end{cases} $$

Then, adding the above three inequalities and by (20), (21), we get

$$\begin{aligned}& \theta(x)-\theta\bigl(\hat{x}^{k}\bigr)+\bigl(w-\hat{w}^{k} \bigr)^{\top}\left\{ \left ( \textstyle\begin{array}{@{}c@{}}-A_{1}^{\top}\hat{\lambda}^{k} \\ -A_{2}^{\top}\hat{\lambda}^{k} \\ A_{1}\hat{x}_{1}^{k}+A_{2}\hat{x}_{2}^{k}-b \end{array}\displaystyle \right )\right. \\& \quad {}+\left. \left ( \textstyle\begin{array}{@{}c@{}}R_{1}{(\hat{x}_{1}^{k} -x_{1}^{k})} \\ (2\alpha-1)\beta A_{2}^{\top}(A_{2}\hat{x}_{2}^{k}-A_{2}x_{2}^{k})+(1-\alpha)A_{2}^{\top}(\hat{\lambda}^{k}-\lambda^{k})/\alpha+R_{2}{(\hat{x}_{2}^{k} -x_{2}^{k})} \\ (1-\alpha)(A_{2}\tilde{x}_{2}^{k}-A_{2}x_{2}^{k})/\alpha+({\lambda}^{k+1}-\lambda ^{k})/(\alpha\beta) \end{array}\displaystyle \right )\right\}\geq0. \end{aligned}$$

Then by (19), we obtain

$$\begin{aligned}& \theta(x)-\theta\bigl(\hat{x}^{k}\bigr)+\bigl(w-\hat{w}^{k} \bigr)^{\top}\left\{ F\bigl(\hat{w}^{k}\bigr)\vphantom{\left ( \textstyle\begin{array}{@{}c@{}} R_{1}(\hat{x}_{1}^{k} -x_{1}^{k}) \\ (2\alpha-1)\beta A_{2}^{\top}(A_{2}\tilde{x}_{2}^{k}-A_{2}x_{2}^{k})+(1-\alpha )A_{2}^{\top}(\hat{\lambda}^{k}-\lambda^{k})/\alpha+R_{2}{(\hat{x}_{2}^{k} -x_{2}^{k})} \\ -(A_{2}\hat{x}_{2}^{k}-A_{2}x_{2}^{k})+(\hat{\lambda}^{k}-\lambda^{k})/(\alpha\beta) \end{array}\displaystyle \right )}\right. \\& \quad {}+\left. \left ( \textstyle\begin{array}{@{}c@{}} R_{1}(\hat{x}_{1}^{k} -x_{1}^{k}) \\ (2\alpha-1)\beta A_{2}^{\top}(A_{2}\tilde{x}_{2}^{k}-A_{2}x_{2}^{k})+(1-\alpha )A_{2}^{\top}(\hat{\lambda}^{k}-\lambda^{k})/\alpha+R_{2}{(\hat{x}_{2}^{k} -x_{2}^{k})} \\ -(A_{2}\hat{x}_{2}^{k}-A_{2}x_{2}^{k})+(\hat{\lambda}^{k}-\lambda^{k})/(\alpha\beta) \end{array}\displaystyle \right )\right\}\geq0. \end{aligned}$$

Then, by (11) (the definition of *Q*), the above inequality can be rewritten as

23 $$ \theta(x)-\theta\bigl(\hat{x}^{k}\bigr)+\bigl(w-\hat {w}^{k}\bigr)^{\top}F\bigl(\hat{w}^{k}\bigr)\geq \bigl(w-\hat{w}^{k}\bigr)^{\top}Q\bigl(w^{k}- \hat{w}^{k}\bigr), $$

for any $w\in\mathcal{W}$. Therefore, if $R_{i}x_{i}^{k}=R_{i}{x}_{i}^{k+1}$ ($i=1,2$), $A_{2}x_{2}^{k}=A_{2}{x}_{2}^{k+1}$ and $\lambda^{k}={\lambda}^{k+1}$, then by (20), we have $\lambda^{k+1}=\hat{\lambda}^{k}$. Then $\hat{\lambda}^{k}=\lambda^{k}$. Thus, we have

$$ Q\bigl(w^{k}-\hat{w}^{k}\bigr)=0 , $$

which together with (23) implies that

$$\theta(x)-\theta\bigl(\hat{x}^{k}\bigr)+\bigl(w-\hat{w}^{k} \bigr)^{\top}F\bigl(\hat{w}^{k}\bigr)\geq0,\quad \forall w\in \mathcal{W}. $$

This indicates that the vector $\hat{w}^{k}$ is a solution of $\operatorname{VI}(\mathcal {W},F,\theta)$. This completes the proof. □

#### Lemma 3.3

*Let* $\{w^{k}\}$ *and* $\{\hat{w}^{k}\}$ *be two sequences generated by SGADMM*. *Then*, *for any* $w\in\mathcal{W}$, *we have*

24 $$ \bigl(w-\hat{w}^{k}\bigr)^{\top}Q \bigl(w^{k}-\hat{w}^{k}\bigr)\geq\frac {1}{2}\bigl( \bigl\Vert w-w^{k+1} \bigr\Vert ^{2}_{H}- \bigl\Vert w-w^{k} \bigr\Vert _{H}^{2}\bigr)+ \frac{\alpha-1}{2\alpha} \bigl\Vert w^{k}-{w}^{k+1} \bigr\Vert _{H}^{2}. $$

#### Proof

Applying the identity

$$(a-b)^{\top}H(c-d)=\frac{1}{2}\bigl(\|a-d\|_{H}^{2}- \|a-c\|_{H}^{2}\bigr)+\frac{1}{2}\bigl(\| c-b \|_{H}^{2}-\|d-b\|_{H}^{2}\bigr), $$

with

$$ a=w,\qquad b=\hat{w}^{k},\qquad c=w^{k}, \qquad d=w^{k+1} , $$

we obtain

$$\begin{aligned} \bigl(w-\hat{w}^{k}\bigr)^{\top}H\bigl(w^{k}-w^{k+1} \bigr) =&\frac{1}{2}\bigl( \bigl\Vert w-w^{k+1} \bigr\Vert _{H}^{2}- \bigl\Vert w-w^{k} \bigr\Vert _{H}^{2}\bigr) \\ &{}+\frac{1}{2}\bigl( \bigl\Vert w^{k}-\hat{w}^{k} \bigr\Vert _{H}^{2}- \bigl\Vert w^{k+1}-\hat{w}^{k} \bigr\Vert _{H}^{2}\bigr). \end{aligned}$$

This together with (12) and (22) implies that

25 $$\begin{aligned} \bigl(w-\hat{w}^{k}\bigr)^{\top}Q \bigl(w^{k}-\hat{w}^{k}\bigr) =&\frac{1}{2}\bigl( \bigl\Vert w-w^{k+1} \bigr\Vert _{H}^{2}- \bigl\Vert w-w^{k} \bigr\Vert _{H}^{2}\bigr) \\ &{}+ \frac{1}{2}\bigl( \bigl\Vert w^{k}-\hat{w}^{k} \bigr\Vert _{H}^{2}- \bigl\Vert w^{k+1}- \hat{w}^{k} \bigr\Vert _{H}^{2}\bigr). \end{aligned}$$

Now let us deal with the last term in (25), which can be written as

$$\begin{aligned}& \bigl\Vert w^{k}-\hat{w}^{k} \bigr\Vert _{H}^{2}- \bigl\Vert w^{k+1}- \hat{w}^{k} \bigr\Vert _{H}^{2} \\& \quad = \bigl\Vert w^{k}-\hat{w}^{k} \bigr\Vert _{H}^{2}- \bigl\Vert \bigl(w^{k}- \hat{w}^{k}\bigr)-\bigl(w^{k}-w^{k+1}\bigr) \bigr\Vert _{H}^{2} \\& \quad = \bigl\Vert w^{k}-\hat{w}^{k} \bigr\Vert _{H}^{2}- \bigl\Vert \bigl(w^{k}- \hat{w}^{k}\bigr)-M\bigl(w^{k}-\hat{w}^{k}\bigr) \bigr\Vert _{H}^{2}\quad (\mbox{using (22)}) \\& \quad = 2\bigl(w^{k}-\hat{w}^{k}\bigr)^{\top}HM \bigl(w^{k}-\hat{w}^{k}\bigr)-\bigl(w^{k}- \hat{w}^{k}\bigr)^{\top}M^{\top}HM \bigl(w^{k}-\hat{w}^{k}\bigr) \\& \quad = \bigl(w^{k}-\hat{w}^{k}\bigr) \bigl(Q^{\top}+Q-M^{\top}HM\bigr) \bigl(w^{k}-\hat{w}^{k}\bigr) \\& \quad \geq \frac{\alpha-1}{2\alpha}\bigl(w^{k}-\hat{w}^{k} \bigr)^{\top}M^{\top}HM\bigl(w^{k}-\hat {w}^{k}\bigr) \quad (\mbox{using (13)}) \\& \quad = \frac{\alpha-1}{2\alpha} \bigl\Vert w^{k}-w^{k+1} \bigr\Vert ^{2}_{H} \quad (\mbox{using (22)}). \end{aligned}$$

Substituting the above inequality into (25), the assertion of this lemma is proved. □

#### Theorem 3.1

*Let* $\{w^{k}\}$ *and* $\{\hat{w}^{k}\}$ *be two sequences generated by SGADMM*. *Then*, *for any* $w\in\mathcal{W}$, *we have*

26 $$\begin{aligned}& \theta(x)-\theta\bigl(\hat{x}^{k}\bigr)+\bigl(w-\hat {w}^{k}\bigr)^{\top}F({w}) \\& \quad \geq\frac{1}{2}\bigl( \bigl\Vert w-w^{k+1} \bigr\Vert ^{2}_{H}- \bigl\Vert w-w^{k} \bigr\Vert _{H}^{2}\bigr)+ \frac {\alpha-1}{2\alpha} \bigl\Vert w^{k}-{w}^{k+1} \bigr\Vert _{H}^{2}. \end{aligned}$$

#### Proof

First, combining (23) and (24), we get

$$\begin{aligned}& \theta(x)-\theta\bigl(\hat{x}^{k}\bigr)+\bigl(w-\hat{w}^{k} \bigr)^{\top}F\bigl(\hat{w}^{k}\bigr) \\& \quad \geq\frac {1}{2} \bigl( \bigl\Vert w-w^{k+1} \bigr\Vert ^{2}_{H}- \bigl\Vert w-w^{k} \bigr\Vert _{H}^{2}\bigr)+ \frac{\alpha-1}{2\alpha} \bigl\Vert w^{k}-{w}^{k+1} \bigr\Vert _{H}^{2}. \end{aligned}$$

From the monotonicity of $F(\cdot)$, we have

$$ \bigl(w-\hat{w}^{k}\bigr)^{\top}\bigl(F(w)-F\bigl( \hat{w}^{k}\bigr)\bigr)\geq0 . $$

Adding the above two inequalities, we obtain the assertion (26). The proof is completed. □

With the above theorem in hand, we are ready to establish the global convergence of SGADMM for solving $\operatorname{VI}(\mathcal{W},F,\theta)$.

#### Theorem 3.2

*Let* $\{w^{k}\}$ *be the sequence generated by SGADMM*. *If* $\alpha>1$, $R_{i}+\beta A_{i}^{\top}A_{i}\succ0$ ($i=1,2$), *then the corresponding sequence* $\{w^{k}\}$ *converges to some* $w^{\infty}$, *which belongs to* $\mathcal{W}^{*}$.

#### Proof

Setting $w=w^{*}$ in (26), we have

$$\begin{aligned}& \bigl\Vert w^{k}-w^{*} \bigr\Vert _{H}^{2}- \frac{\alpha-1}{\alpha} \bigl\Vert w^{k}-{w}^{k+1} \bigr\Vert _{H}^{2} \\& \quad \geq 2\bigl\{ \theta\bigl(\hat{x}^{k}\bigr)-\theta\bigl(x^{*} \bigr)+\bigl(\hat{w}^{k}-w^{*}\bigr)^{\top}F\bigl({w^{*}}\bigr) \bigr\} + \bigl\Vert w^{k+1}-w^{*} \bigr\Vert ^{2}_{H} \\& \quad \geq \bigl\Vert w^{k+1}-w^{*} \bigr\Vert ^{2}_{H}, \end{aligned}$$

where the second inequality follows from $w^{*}\in\mathcal{W}^{*}$. Thus, we have

27 $$ \bigl\Vert w^{k+1}-w^{*} \bigr\Vert ^{2}_{H}\leq \bigl\Vert w^{k}-w^{*} \bigr\Vert _{H}^{2}-\frac {\alpha-1}{\alpha} \bigl\Vert w^{k}-{w}^{k+1} \bigr\Vert _{H}^{2}. $$

Summing over $k=0,1,\ldots,\infty$, it yields

$$\sum_{k=0}^{\infty}\bigl\Vert w^{k}-w^{k+1} \bigr\Vert ^{2}_{H}\leq \frac{\alpha}{\alpha-1} \bigl\Vert w^{0}-w^{*} \bigr\Vert _{H}^{2}. $$

By $\alpha>1$ and the positive semi-definite of *H*, the above inequality implies that

$$\lim_{k\rightarrow\infty} \bigl\Vert w^{k}-w^{k+1} \bigr\Vert ^{2}_{H}=0. $$

Thus, by the definition of *H*, we have

28 $$ \lim_{k\rightarrow\infty} \bigl\Vert x_{1}^{k}-x_{1}^{k+1} \bigr\Vert ^{2}_{R_{1}}=\lim_{k\rightarrow\infty} \bigl\Vert v^{k}-v^{k+1} \bigr\Vert ^{2}_{H_{1}}=0, $$

where

$$H_{1}=\left ( \textstyle\begin{array}{@{}c@{\quad}c@{}} R_{2}+\frac{2\alpha^{2}-2\alpha+1}{\alpha}\beta A_{2}^{\top}A_{2}&\frac{1-\alpha }{\alpha}A_{2}^{\top}\\ \frac{1-\alpha}{\alpha}A_{2}&\frac{1}{\alpha\beta}I_{l} \end{array}\displaystyle \right ), $$

is positive definite by $R_{2}+\beta A_{2}^{\top}A_{2}\succ0$. From (27) again, we have

$$\bigl\Vert w^{k+1}-w^{*} \bigr\Vert ^{2}_{H}\leq \bigl\Vert w^{0}-w^{*} \bigr\Vert ^{2}_{H}, $$

which indicates that the sequence $\{Hw^{k}\}$ is bounded. Thus, $\{ R_{1}x_{1}^{k}\}_{k=0}^{\infty}$ and $\{H_{1}v^{k}\}_{k=0}^{\infty}$ are both bounded. Then $\{v^{k}\}_{k=0}^{\infty}$ is bounded. If $R_{1}\succ0$, $\{x_{1}^{k}\} _{k=0}^{\infty}$ is bounded; otherwise, $A_{1}^{\top}A_{1}\succ0$, that is, $A_{1}$ is full-column rank, which together with $A_{1}x_{1}=(\lambda ^{k}-\lambda^{k+1})/(\alpha\beta)+(1-\alpha)(A_{2}x_{2}^{k}-b)/\alpha -(A_{2}x_{2}^{k+1}-b)/\alpha$ implies that $\{x_{1}^{k}\}_{k=0}^{\infty}$ is bounded. In conclusion, $\{w^{k}\} _{k=0}^{\infty}$ is bounded.

Then, from (28) and $H_{1}\succ0$, the sequence $\{v^{k}\}$ is convergent. Suppose it converges to $v^{\infty}$. Let $w^{\infty}=(x_{1}^{\infty},v^{\infty})$ be a cluster point of $\{w^{k}\}$ and $\{w^{k_{j}}\} $ be the corresponding subsequence. On the other hand, by (20) and (28), we have

$$\lim_{k\rightarrow\infty}R_{1}\bigl(x_{1}^{k}- \hat{x}_{1}^{k}\bigr)=0,\qquad \lim_{k\rightarrow \infty} \bigl(x_{2}^{k}-\hat{x}_{2}^{k}\bigr)=0 $$

and

$$\lim_{k\rightarrow\infty}\bigl(\lambda^{k}-\hat{ \lambda}^{k}\bigr)=\lim_{k\rightarrow \infty}\bigl( \lambda^{k}-\lambda^{k+1}+\beta\bigl(A_{2} \hat{x}_{2}^{k}-A_{2}x_{2}^{k} \bigr)\bigr)=0. $$

Thus,

29 $$ \lim_{k\rightarrow\infty}Q\bigl(w^{k}- \hat{w}^{k}\bigr)=0. $$

Then, taking the limit along the subsequence $\{w^{k_{j}}\}$ in (23) and using (29), for any $w\in\mathcal{W}$, we obtain

$$\theta(x)-\theta\bigl(x^{\infty}\bigr)+\bigl(w-w^{\infty}\bigr)^{\top}F\bigl(w^{\infty}\bigr)\geq0, $$

which indicates that $w^{\infty}$ is a solution of $\operatorname{VI}(\mathcal {W},F,\theta)$. Then, since $w^{*}$ in (27) is arbitrary, we can set $w^{*}=w^{\infty}$ and conclude that the whole generated sequence $\{w^{k}\}$ converges by $R_{i}+\beta A_{i}^{\top}A_{i}\succ0$ ($i=1,2$). This completes the proof. □

### Convergence rate

Now, we are going to prove the worst-case $\mathcal{O}(1/t)$ convergence rate of SGADMM in both the ergodic and the non-ergodic senses.

#### Theorem 3.3

*Let* $\{w^{k}\}$ *and* $\{\hat{w}^{k}\}$ *be the sequences generated by SGADMM*, *and set*

$$\bar{w}^{t}=\frac{1}{t+1}\sum_{k=0}^{t} \hat{w}^{k}. $$

*Then*, *for any integer* $t\geq0$, *we have* $\bar{w}^{t}\in\mathcal{W}$, *and*

30 $$ \theta(\bar{x}_{t})-\theta(x)+(\bar {w}_{t}-w)^{\top}F(w)\leq\frac{1}{2(t+1)} \bigl\Vert w-w^{0} \bigr\Vert _{H}^{2},\quad \forall w\in \mathcal{W}. $$

#### Proof

From (17) and the convexity of the set $\mathcal{W}$, we have $\bar{w}^{k}\in\mathcal{W}$. From (26), we have

$$\theta(x)-\theta\bigl(\hat{x}^{k}\bigr)+\bigl(w-\hat{w}^{k} \bigr)^{\top}F({w})+\frac{1}{2} \bigl\Vert w-w^{k} \bigr\Vert _{H}^{2}\geq\frac{1}{2} \bigl\Vert w-w^{k+1} \bigr\Vert ^{2}_{H},\quad \forall w\in \mathcal{W}. $$

Summing the above inequality over $k=0,1,\ldots,t$, we get

$$(t+1)\theta(x)-\sum_{k=0}^{t}\theta\bigl( \hat{x}^{k}\bigr)+ \Biggl( (t+1)w-\sum_{k=0}^{t} \hat{w}^{k} \Biggr)^{\top}F({w})+\frac{1}{2} \bigl\Vert w-w^{0} \bigr\Vert _{H}^{2}\geq0,\quad \forall w \in\mathcal{W}. $$

By the definition of $\bar{w}^{t}$ and the convexity of $\theta(\cdot)$, the assertion (30) follows immediately from the above inequality. This completes the proof. □

The proof of the next two lemmas is referred to those of Lemmas 5.1 and 5.2 in [24]. For completeness, we give the detail proof.

#### Lemma 3.4

*Let* $\{w^{k}\}$ *be the sequence generated by SGADMM*. *Then we have*

31 $$\begin{aligned}& \bigl(w^{k}-w^{k+1}\bigr)^{\top}H\bigl\{ \bigl(w^{k}-w^{k+1}\bigr)-\bigl(w^{k+1}-{w}^{k+2} \bigr)\bigr\} \\& \quad \geq\frac{3\alpha-1}{4\alpha} \bigl\Vert \bigl(w^{k}-w^{k+1} \bigr)-\bigl(w^{k+1}-{w}^{k+2}\bigr) \bigr\Vert _{H}^{2}. \end{aligned}$$

#### Proof

Setting $w=\hat{w}^{k+1}$ in (23), we have

$$\theta\bigl(\hat{x}^{k+1}\bigr)-\theta\bigl(\hat{x}^{k} \bigr)+\bigl(\hat{w}^{k+1}-\hat{w}^{k}\bigr)^{\top}F \bigl(\hat{w}^{k}\bigr)\geq\bigl(\hat{w}^{k+1}- \hat{w}^{k}\bigr)^{\top}Q\bigl(w^{k}- \hat{w}^{k}\bigr). $$

Similarly setting $w=\hat{w}^{k}$ in (23) for $k:=k+1$, we get

$$\theta\bigl(\hat{x}^{k}\bigr)-\theta\bigl(\hat{x}^{k+1} \bigr)+\bigl(\hat{w}^{k}-\hat {w}^{k+1}\bigr)^{\top}F \bigl(\hat{w}^{k+1}\bigr)\geq\bigl(\hat{w}^{k}- \hat{w}^{k+1}\bigr)^{\top}Q\bigl(w^{k+1}- \hat{w}^{k+1}\bigr). $$

Then, adding the above two inequalities and using the monotonicity of the mapping $F(\cdot)$, we get

32 $$ \bigl(\hat{w}^{k}-\hat{w}^{k+1} \bigr)^{\top}Q\bigl\{ \bigl(w^{k}-\hat {w}^{k}\bigr)- \bigl(w^{k+1}-\hat{w}^{k+1}\bigr)\bigr\} \geq0. $$

By (32), we have

$$\begin{aligned}& \bigl({w}^{k}-{w}^{k+1}\bigr)^{\top}Q\bigl\{ \bigl(w^{k}-\hat{w}^{k}\bigr)-\bigl(w^{k+1}- \hat{w}^{k+1}\bigr)\bigr\} \\& \quad = \bigl\{ \bigl(w^{k}-\hat{w}^{k}\bigr)- \bigl(w^{k+1}-\hat{w}^{k+1}\bigr)+\bigl(\hat{w}^{k}- \hat{w}^{k+1}\bigr)\bigr\} ^{\top}Q\bigl\{ \bigl(w^{k}- \hat{w}^{k}\bigr)-\bigl(w^{k+1}-\hat{w}^{k+1}\bigr) \bigr\} \\& \quad = \bigl\Vert \bigl(w^{k}-\hat{w}^{k}\bigr)- \bigl(w^{k+1}-\hat{w}^{k+1}\bigr) \bigr\Vert _{Q}^{2}+\bigl(\hat{w}^{k}-\hat {w}^{k+1} \bigr)^{\top}Q\bigl\{ \bigl(w^{k}-\hat{w}^{k}\bigr)- \bigl(w^{k+1}-\hat{w}^{k+1}\bigr)\bigr\} \\& \quad \geq \bigl\Vert \bigl(w^{k}-\hat{w}^{k}\bigr)- \bigl(w^{k+1}-\hat{w}^{k+1}\bigr) \bigr\Vert _{Q}^{2}. \end{aligned}$$

Using (13), (22) and $Q=HM$ on both sides of the above inequality, we get

$$\begin{aligned}& \bigl(w^{k}-w^{k+1}\bigr)^{\top}H\bigl\{ \bigl(w^{k}-w^{k+1}\bigr)-\bigl(w^{k+1}-{w}^{k+2} \bigr)\bigr\} \\& \quad = \bigl(w^{k}-w^{k+1}\bigr)^{\top}QM^{-1}\bigl\{ \bigl(w^{k}-w^{k+1}\bigr)- \bigl(w^{k+1}-{w}^{k+2}\bigr)\bigr\} \\& \quad = \bigl(w^{k}-w^{k+1}\bigr)^{\top}Q\bigl\{ \bigl(w^{k}-\hat{w}^{k}\bigr)-\bigl(w^{k+1}- \hat{w}^{k+1}\bigr)\bigr\} \\& \quad \geq \bigl\Vert \bigl(w^{k}-\hat{w}^{k}\bigr)- \bigl(w^{k+1}-\hat{w}^{k+1}\bigr) \bigr\Vert _{Q}^{2} \\& \quad = \bigl[\bigl(w^{k}-\hat{w}^{k}\bigr)- \bigl(w^{k+1}-\hat{w}^{k+1}\bigr)\bigr]^{\top}Q\bigl[ \bigl(w^{k}-\hat {w}^{k}\bigr)-\bigl(w^{k+1}- \hat{w}^{k+1}\bigr)\bigr] \\& \quad = \bigl[\bigl(w^{k}-w^{k+1}\bigr)-\bigl(w^{k+1}-w^{k+2} \bigr)\bigr]^{\top}M^{-1}QM^{-1}\bigl[ \bigl(w^{k}-w^{k+1}\bigr)-\bigl(w^{k+1}-w^{k+2} \bigr)\bigr] \\& \quad \geq \frac{3\alpha-1}{4\alpha}\bigl[\bigl(w^{k}-w^{k+1}\bigr)- \bigl(w^{k+1}-w^{k+2}\bigr)\bigr]^{\top}\\& \qquad {}\times M^{-1}MHMM^{-1}\bigl[\bigl(w^{k}-w^{k+1} \bigr)-\bigl(w^{k+1}-w^{k+2}\bigr)\bigr] \\& \quad = \frac{3\alpha-1}{4\alpha} \bigl\Vert \bigl(w^{k}-w^{k+1} \bigr)-\bigl(w^{k+1}-w^{k+2}\bigr) \bigr\Vert _{H}^{2}. \end{aligned}$$

Then we get the assertion (31). The proof is completed. □

#### Lemma 3.5

*Let* $\{w^{k}\}$ *be the sequence generated by SGADMM*. *Then we have*

33 $$ \bigl\Vert w^{k+1}-{w}^{k+2} \bigr\Vert ^{2}_{H}\leq \bigl\Vert w^{k}-{w}^{k+1} \bigr\Vert ^{2}_{H}-\frac{\alpha-1}{2\alpha} \bigl\Vert \bigl(w^{k}-w^{k+1}\bigr)-\bigl(w^{k+1}-{w}^{k+2} \bigr) \bigr\Vert _{H}^{2}. $$

#### Proof

Setting $a:=(w^{k}-{w}^{k+1})$ and $b:=(w^{k+1}-{w}^{k+2})$ in the identity

$$\|a\|_{H}^{2}-\|b\|_{H}^{2}=2a^{\top}H(a-b)-\|a-b\|_{H}^{2}, $$

we can derive

$$\begin{aligned}& \bigl\Vert w^{k}-{w}^{k+1} \bigr\Vert _{H}^{2}- \bigl\Vert w^{k+1}-{w}^{k+2} \bigr\Vert _{H}^{2} \\& \quad = 2\bigl(w^{k}-{w}^{k+1}\bigr)^{\top}H\bigl\{ \bigl(w^{k}-{w}^{k+1}\bigr)-\bigl(w^{k+1}-{w}^{k+2} \bigr)\bigr\} - \bigl\Vert \bigl(w^{k}-{w}^{k+1}\bigr)- \bigl(w^{k+1}-{w}^{k+2}\bigr) \bigr\Vert _{H}^{2} \\& \quad \geq \frac{3\alpha-1}{2\alpha} \bigl\Vert \bigl(w^{k}-w^{k+1} \bigr)-\bigl(w^{k+1}-{w}^{k+2}\bigr) \bigr\Vert _{H}^{2}- \bigl\Vert \bigl(w^{k}-{w}^{k+1} \bigr)-\bigl(w^{k+1}-{w}^{k+2}\bigr) \bigr\Vert _{H}^{2} \\& \quad = \frac{\alpha-1}{2\alpha} \bigl\Vert \bigl(w^{k}-w^{k+1} \bigr)-\bigl(w^{k+1}-{w}^{k+2}\bigr) \bigr\Vert _{H}^{2}, \end{aligned}$$

which completes the proof of the lemma. □

Based on Lemma 3.5, now we establish the worst-case $\mathcal{O}(1/t)$ convergence rate of SGADMM in a non-ergodic sense.

#### Theorem 3.4

*Let* $\{w^{k}\}$ *be the sequence generated by SGADMM*. *Then*, *for any* $w^{*}\in\mathcal{W}^{*}$ *and integer* $t\geq0$, *we have*

34 $$ \bigl\Vert w^{t}-w^{t+1} \bigr\Vert _{H}^{2}\leq\frac{\alpha }{(t+1)(\alpha-1)} \bigl\Vert w^{0}-w^{*} \bigr\Vert _{H}^{2}. $$

#### Proof

By (27), we get

$$\frac{\alpha-1}{\alpha}\sum_{k=0}^{t} \bigl\Vert w^{k}-w^{k+1} \bigr\Vert ^{2}_{H} \leq \bigl\Vert w^{0}-w^{*} \bigr\Vert _{H}^{2}. $$

This and (33) imply that

$$\frac{(t+1)(\alpha-1)}{\alpha} \bigl\Vert w^{t}-{w}^{t+1} \bigr\Vert _{H}^{2}\leq \bigl\Vert w^{0}-w^{*} \bigr\Vert _{H}^{2}. $$

Therefore, the assertion of this theorem comes from the above inequality immediately. The proof is completed. □

#### Remark 3.2

From (34), we see that the larger *α* is, the smaller $\frac{\alpha}{\alpha-1}$, which controls the upper bounds of $\|w^{t}-w^{t+1}\|_{H}^{2}$. Therefore, it seems that larger values of *α* are more beneficial for speeding up the convergence of SGADMM.

## Numerical experiments

In this section, we present some numerical experiments to verify the efficiency of SGADMM for solving compressed sensing. Those numerical experiments are performed in Matlab R2010a on a ThinkPad computer equipped with Windows XP, 997 MHz and 2 GB of memory.

Compressed sensing (CS) is to recover a sparse signal $\bar{x}\in \mathcal{R}^{n}$ from an undetermined linear system $b=A\bar{x}$, where $A\in\mathcal{R}^{m\times n}$ ($m\ll n$), can be depicted as problem (2).

Obviously, Problem (2) is equivalent to the following two models:

- Model 1: Problem (3).
- Model 2:
  35 $$ \begin{aligned} &\min\mu\|x_{1} \|_{1}+\frac{1}{2}\|Ax_{2}-y\|^{2} \\ &\quad \mbox{s.t. } {-}x_{1}-x_{2}=0, \\ &\hphantom{\quad \mbox{s.t.}}\ x_{1}\in\mathcal{R}^{n}, x_{2} \in\mathcal{R}^{n}. \end{aligned} $$

### The iterative schemes for (3) and (35)

Since (3) and (35) are both some concrete models of (1), SGADMM are applicable to them. Below, we elaborate on how to derive the closed-form solutions for the sub-problems resulting by SGADMM.

For problem (3), its first two sub-problems resulting by SGADMM are as follows.

• With the given $x_{2}^{k}$ and $\lambda^{k}$, the $x_{1}$-sub-problem in (17) is (here $R_{1}=0$)

$$x_{1}^{k+1}=\mathop{\operatorname{argmin}}_{x_{1}\in\mathcal{R}^{n}} \biggl\{ \frac{1}{2} \Vert x_{1} \Vert _{2}^{2}+x_{1}^{\top}\lambda+\frac{\alpha\beta}{2} \bigl\Vert x_{1}-Ax_{2}^{k}+y \bigr\Vert ^{2}\biggr\} , $$

which has the following closed-form solution:

$$x_{1}^{k+1}=\frac{1}{1+\alpha\beta}\bigl(\alpha\beta \bigl(Ax_{2}^{k}-y\bigr)-\lambda^{k}\bigr). $$

• With the updated $x_{1}^{k+1}$, the $x_{2}$-sub-problem in (17) is (here $R_{2}=\tau I_{n}-(2\alpha-1)\beta A^{\top}A$ with $\tau \geq(2\alpha-1)\beta\|A^{\top}A\|$)

$$x_{2}^{k+1}=\mathop{\operatorname{argmin}}_{x_{2}\in\mathcal{R}^{n}} \biggl\{ \mu \Vert x_{2} \Vert _{1}-x_{2}^{\top}A^{\top}\lambda^{k}+\frac{(2\alpha-1)\beta}{2} \bigl\Vert -x_{1}^{k+1}+Ax_{2}-y \bigr\Vert ^{2}+ \frac{1}{2} \bigl\Vert x_{2}-x_{2}^{k} \bigr\Vert ^{2}_{R_{2}}\biggr\} , $$

and its closed-form solution is given by

$$x_{2}^{k+1}=\operatorname{shrink}_{\frac{\mu}{\tau}}\bigl((2 \alpha-1)\beta A^{\top}\bigl({x_{1}^{k+1}}+y\bigr)/ \tau+\bigl(\tau I_{n}-(2\alpha-1)\beta A^{\top}A \bigr)x_{2}^{k}/\tau +A^{\top}\lambda^{k}/ \tau\bigr), $$

where, for any $c>0$, $\operatorname{shrink}_{c}(\cdot)$ is defined as

$$\operatorname{shrink}_{c}(g):=g-\min\bigl\{ c,|g|\bigr\} \frac{g}{|g|},\quad \forall g\in \mathcal{R}^{n}, $$

and $(g/|g|)_{i}$ should be taken 0 if $|g|_{i}=0$.

Similarly, for problem (35), its first two sub-problems resulting by SGADMM are as follows.

• With the given $x_{2}^{k}$ and $\lambda^{k}$, the $x_{1}$-sub-problem in (17) is (here $R_{1}=0$)

$$x_{1}^{k+1}=\mathop{\operatorname{argmin}}_{x_{1}\in\mathcal{R}^{n}} \biggl\{ \mu \Vert x_{1} \Vert _{1}+\frac {\alpha\beta}{2} \biggl\Vert x_{1}-\biggl(x_{2}^{k}+ \frac{1}{\alpha\beta}\lambda^{k}\biggr) \biggr\Vert ^{2}\biggr\} , $$

and its closed-form solution is given by

$$x_{1}^{k+1}=\operatorname{shrink}_{\frac{\mu}{\alpha\beta}} \biggl(x_{2}^{k}+\frac {1}{\alpha\beta}\lambda^{k} \biggr). $$

• With the updated $x_{1}^{k+1}$, the $x_{2}$-sub-problem in (17) is (here $R_{2}=\tau I_{n}-A^{\top}A$ with $\tau\geq\|A^{\top}A\|$)

$$x_{2}^{k+1}=\mathop{\operatorname{argmin}}_{x_{2}\in\mathcal{R}^{n}} \biggl\{ \frac{1}{2} \Vert Ax_{2}-y \Vert ^{2}+x_{2}^{\top}\lambda^{k}+\frac{(2\alpha-1)\beta}{2} \bigl\Vert x_{2}-x_{1}^{k+1} \bigr\Vert ^{2}+\frac {1}{2} \bigl\Vert x_{2}-x_{2}^{k} \bigr\Vert ^{2}_{R_{2}}\biggr\} , $$

and its closed-form solution is given by

$$x_{2}^{k+1}=\frac{1}{\tau+(2\alpha-1)\beta}\bigl(A^{\top}y- \lambda^{k}+(2\alpha -1)\beta x_{1}^{k+1}+A^{\top}Ax_{2}^{k}\bigr). $$

Obviously, the above two iterative schemes both need to compute $A^{\top}A$ and $A^{\top}y$, which is quite time consuming if *n* is large. However, noting that these two terms are invariant during the iteration process, therefore we need only compute them once before all iterations.

Regarding the penalty parameter *β* and the constant *α* in SGADMM, any $\beta>0$ and $\alpha\geq1$ can ensure the convergence of SGADMM in theory. There are two traditional methods to determine them in practice. One is the tentative method, which is easy to execute. The other is the self-adaptive adjustment method, which needs much computation. In this experiment, for *β* and *α*, we use the tentative method to determine their suitable values. For *β*, Xiao *et al.* [35] set $\beta=\mathtt{mean}(\mathtt{abs}(y))$ for ADMM. Motivated by this choice, we set $\beta=\mathtt{mean}(\mathtt{abs}(y))/(2\alpha -1)$ in our algorithm. As for the parameter *α*, we have pointed out in Remark 3.2 that larger values of *α* may be beneficial for our algorithm. Here, we use (3) to do a little experiment to test this. We choose different values of *α* in the interval $[1, 2]$. Specifically, we choose $\alpha\in\{1.0, 1.1, \ldots , 2\}$. Other data about this experiment are as follows: the proximal parameter *τ* is set as $\tau=1.01(2\alpha-1)\beta\|A^{\top}A\|$; the observed signal *y* is set as $y=Ax+0.01\times\mathtt{randn}(m,1)$ in Matlab; the sensing matrix *A* and the original signal *x* are generated by

$$\bar{A}=\mathtt{randn}(m,n),\qquad [Q, R]=\mathtt{qr}\bigl(\bar{A}',0 \bigr),\qquad A = Q', $$

and

$$x = \mathtt{zeros}(n,1); \qquad p =\mathtt{randperm}(n);\qquad x\bigl(p(1:k) \bigr) = \mathtt {randn}(k,1). $$

Then the observed signal *y* is further set as $(R^{\top})^{-1}y$. The initial points are set as $x_{2}^{0}=A^{\top}y$, $\lambda^{0}=Ax_{2}^{0}$. In addition, we set the regularization parameter $\mu=0.01$, and the dimensions of the problem are set as $n=1\text{,}000$, $m = 300$, $k = 60$, where *k* denotes the number of the non-zeros in the original signal *x*. To evaluate the quality of the recovered signal, let us define the quantity ‘RelErr’ as follows:

$$\operatorname{RelErr}=\frac{\|\tilde{x}-{x}\|}{\|{x}\|}, $$

where *x̃* denotes the recovered signal. The stopping criterion is

$$\frac{\|f_{k}-f_{k-1}\|}{\|f_{k-1}\|}< 10^{-5}, $$

where $f_{k}$ denotes the function value of (2) at the iterate $x_{k}$.

### Numerical results

**Figure 1:**
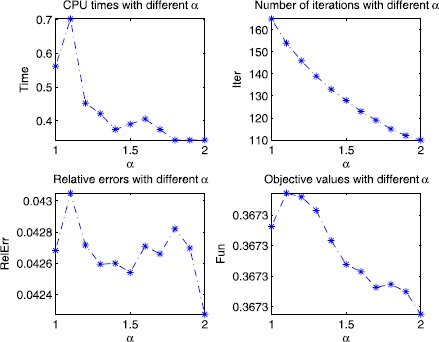
**Sensitivity test on the parameter** ***α*** **.**

The numerical results are graphicly shown in Figure 1. Clearly, the numerical results in Figure 1 indicate that Remark 3.2 is reasonable. Both CPU time and number of iterations are descent with respect to *α*. Then, in the following, we set $\alpha=1.4$, which is a moderate choice for *α*.

**Figure 2:**
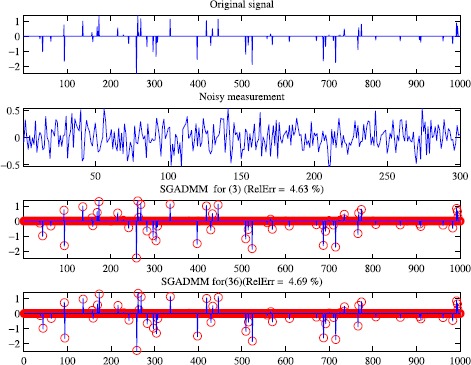
**Numerical results of SGADMM for (** **3** **) and (** **35** **).** The top: the original signal; the second: the noisy measurement; the bottom two: recovered signal.

Now, let us graphically show the recovered results of SGADMM for (3) and (35). The proximal parameter *τ* is set as $\tau=1.01(2\alpha-1)\beta\| A^{\top}A\|$ for (3), and $\tau=1.01\|A^{\top}A\|$ for (35). The initial points are set as $x_{2}^{0}=A^{\top}y$, $\lambda^{0}=Ax_{2}^{0}$ for (3), and $x_{2}^{0}=A^{\top}y$, $\lambda^{0}=x_{2}^{0}$ for (35). Other parameters are set the same as above. Figure 2 reports the numerical results of SGADMM for (3) and (35).

The bottom two subplots in Figure 2 indicate that our new method SGADMM can be used to solve (3) and (35).

In the following, we do some numerical comparisons to illustrate the advantage of our new method and to analyze which one is more suitable to compressed sensing (2) between the two models (3) and (35). SGADMM for (3) is denoted by SGADMM1, SGADMM for (35) is denoted by SGADMM2. We also compare SGADMM with the classical ADMM. The numerical results are listed in Table 1, where ‘Time’ denotes the CPU time (in seconds), and ‘Iter’ denotes the number of iterations required for the whole recovering process, $m = \mathtt{floor}(\gamma n)$, $k = \mathtt{floor}(\sigma m)$. The numerical results are the average of the numerical results of ten runs with different combinations of *γ* and *σ*.

**Table 1:** **Comparison of SGADMM1, SGADMM2 and ADMM**

***n***	***γ***	***σ***	**SGADMM1**			**SGADMM2**			**ADMM**		
			**Time**	**Iter**	**RelErr**	**Time**	**Iter**	**RelErr**	**Time**	**Iter**	**RelErr**
1,000	0.3	0.2	0.6911	92.4	0.0387	0.9578	266.0	0.0394	0.9812	264.0	0.0393
	0.2	0.2	0.6661	118.6	0.0825	1.3915	421.8	0.0915	1.3603	419.6	0.0915
	0.2	0.1	0.5008	85.3	0.0609	0.4758	139.0	0.0579	0.5008	138.0	0.0539
2,000	0.3	0.2	2.1965	90.0	0.0437	3.6535	267.7	0.0447	3.5412	265.6	0.0447
	0.2	0.2	2.2339	109.6	0.0785	5.2182	431.4	0.0874	5.1543	429.0	0.0874
	0.2	0.1	1.5428	79.9	0.0534	1.7893	142.8	0.0467	1.7613	140.8	0.0513

### Discussion

The numerical results in Table 1 indicate that: (1) by the criterion ‘RelErr’, all methods successfully solved all the cases; (2) by the criteria ‘Time’ and ‘Iter’, SGADMM1 performs better than the other two methods. Especially the number of iterations of SGADMM1 is about at most two-thirds of the other two methods. This experiment also indicate that the model (3) is also an effective model for compressed sensing, and sometimes it is more efficient than the model (35), though they are equivalent in theory. In conclusion, by choosing some relaxation factor $\alpha\in[1,+\infty)$, SGADMM may be more efficient than the classical ADMM.

## Conclusions

In this paper, we have proposed a symmetric version of the generalized ADMM (SGADMM), which generalizes the feasible set of the relaxation factor *α* from $(0,2)$ to $[1,+\infty)$. Under the same conditions, we have proved the convergence results of the new method. Some numerical results illustrate that it may perform better than the classical ADMM. In the future, we shall study SGADMM with $\alpha\in (0,1)$ to perfect the theoretical system.
